# Addison’s Disease Symptoms – A Cross Sectional Study in Urban South Africa

**DOI:** 10.1371/journal.pone.0053526

**Published:** 2013-01-07

**Authors:** Ian Louis Ross, Naomi S. Levitt

**Affiliations:** Division of Endocrinology, Groote Schuur Hospital, Department of Medicine, University of Cape Town, Cape Town, South Africa; Tabriz University of Medical Sciences, Islamic Republic of Iran

## Abstract

**Background:**

Addison’s disease is a potentially life-threatening disorder, and prompt diagnosis, and introduction of steroid replacement has resulted in near normal life-expectancy. There are limited data describing the clinical presentation of Addison’s disease in South Africa. It is hypothesised that patients may present in advanced state of ill-health, compared to Western countries.

**Patients:**

A national database of patients was compiled from primary care, referral centres and private practices. 148 patients were enrolled (97 white, 34 mixed ancestry, 5 Asian and 12 black).

**Methods:**

Demographic and clinical data were elicited using questionnaires. Biochemical data were obtained from folder reviews and laboratory archived results.

**Results:**

The majority of the cohort was women (62%). The median and inter-quartile age range (IQR) of patients at enrolment was 46.0 (32.0–61.0) years, with a wide range from 2.8–88.0 years. The median and IQR age at initial diagnosis was 34.0 (20.0–45.0) years (range 0.02–77.0) years, indicating that at the time of enrolment, the patients, on average, were diagnosed with Addison’s disease 12 years previously. Hyperpigmentation was observed in 76%, nausea and vomiting occurred in more than 40%, and weight loss was noted in 25%. Loss of consciousness as a presenting feature was recorded in 20**%.** with a 95% confidence interval [CI] of (14–28%) and shock occurred in 5% CI (1.5–8.5%). Case-finding was recorded at 3.1 per million.

**Conclusions:**

The usual constellation of hyperpigmentation, nausea, vomiting and weight loss suggests Addison’s disease, but a significant proportion present with an advanced state of ill-health and Addisonian crises. A lower prevalence rate, compared to Western countries is suggested.

## Introduction

South Africa has a population of 48 million people. In the absence of national insurance there is an unequal access to health-care, which consists of a vast public health sector and a small, but expanding private health sector [1]. Addison’s disease (primary adrenal insufficiency) results from the destruction of the adrenal cortex, leading to decreased cortisol and aldosterone production. These hormones are vital for survival during stressful situations including infection [2]. This disorder is highly treatable and warrants prompt recognition. The described prevalence in Western countries is 39 to 144 per million [3]–[5]. The study by Ross et al describes the underlying aetiology of Addison’s disease in a cohort of South Africans, which is predominated by autoimmunity [6].

Addison’s disease, like syphilis has been referred to as one of the great mimickers of medicine. It is therefore not surprising that a cross-sectional study from Germany found a significant delay in making the diagnosis, as 20% of the subjects had symptoms for longer than five years prior to being diagnosed [7]. The vast majority of cohort studies of Addison’s disease emanate from Western countries and only isolated reports from Africa describe the clinical presentation [8], [9].

We wished to describe the clinical presentation of Addison’s disease in South Africa, with the hypothesis that the presentation may be similar to Western countries, albeit that the signs and symptoms may be more severe due to the lack of access to health-care. It is expected that a description of the clinical presentation of this disorder will serve to remind clinicians of this disorder so that this diagnosis is not missed.

## Methods

### Patients

A national database of patients with Addison’s disease was compiled from primary care, referral centres and private practices.

### Method of Registry Compilation

Since databases in South Africa were not available for Addison’s disease, a systematic approach was adopted of initially inviting patients attending quaternary hospitals, followed by patients attending tertiary hospitals and private care facilities. This was followed by inviting prospective participants attending both secondary and primary health-care facilities between the years 2005–2010. A private commercial database (MedPages) of medical specialists and general practitioners sent in addition, 9 600 personalised e-mails to all specialist physicians, paediatricians and general practitioners registered with this organisation. As Addison’s disease is designated as a medical condition that enjoys the prescribed minimum benefit, it is a statutory requirement that patients belonging to a medical aid have the total cost of their treatment reimbursed. [10] The medical insurance companies were only able to communicate the names of the treating physicians of Addison’s patients to our research group of those patients registered, in order to prevent a breach of confidentiality. These treating physicians were then requested by letter or e-mail to invite his or her patients to participate in the study.

### Ethics and Informed Consent

Approval to conduct the study was obtained from the Research and Ethics committee of the University of Cape Town, which endorses the latest declaration of Helsinki. Ethics approval was also obtained from the respective research and ethics committees overseeing the various faculties of health sciences including Nelson Mandela School of Medicine, University of KwaZulu-Natal, University of the Free State, University of Stellenbosch, University of Pretoria and the University of Witwatersrand. All participants signed written informed consent.

### Confirmation of Addison’s Disease and Clinical Presentation

The diagnosis of Addison’s disease was made on the basis of a suggestive clinical presentation, low basal cortisol concentration and simultaneously elevated adrenocorticotrophic hormone (ACTH) concentration, or where indicated, a peak cortisol, following 250 µg ACTH stimulation of less than 550 nmol/L, associated with a basal raised plasma ACTH, exceeding 10.1 pmol/L. These criteria have been published previously [11]. Addison’s disease was confirmed in each case by specialist internist, paediatrician or endocrinologist. They were also only considered eligible if they were resident in South Africa. The clinical symptoms of the patients at diagnosis of Addison’s disease were elicited by interviewing them at the time of enrolment and where possible, the clinical details from the notes at the time of presentation were included in the database.

### Statistical Methods

Patient characteristics with non-normal distribution were described using median and interquartile range. Continuous data were compared between groups using the Mann-Whitney and chi-squared tests were used for comparisons of binary and categorical variables. Binomial confidence intervals (CI) were used as a measure of the precision of the estimate. All analyses were conducted were conducted using Stata (TM) version 10.0 (Stata Corp., College Station, Texas).

## Results

**Figure 1:**
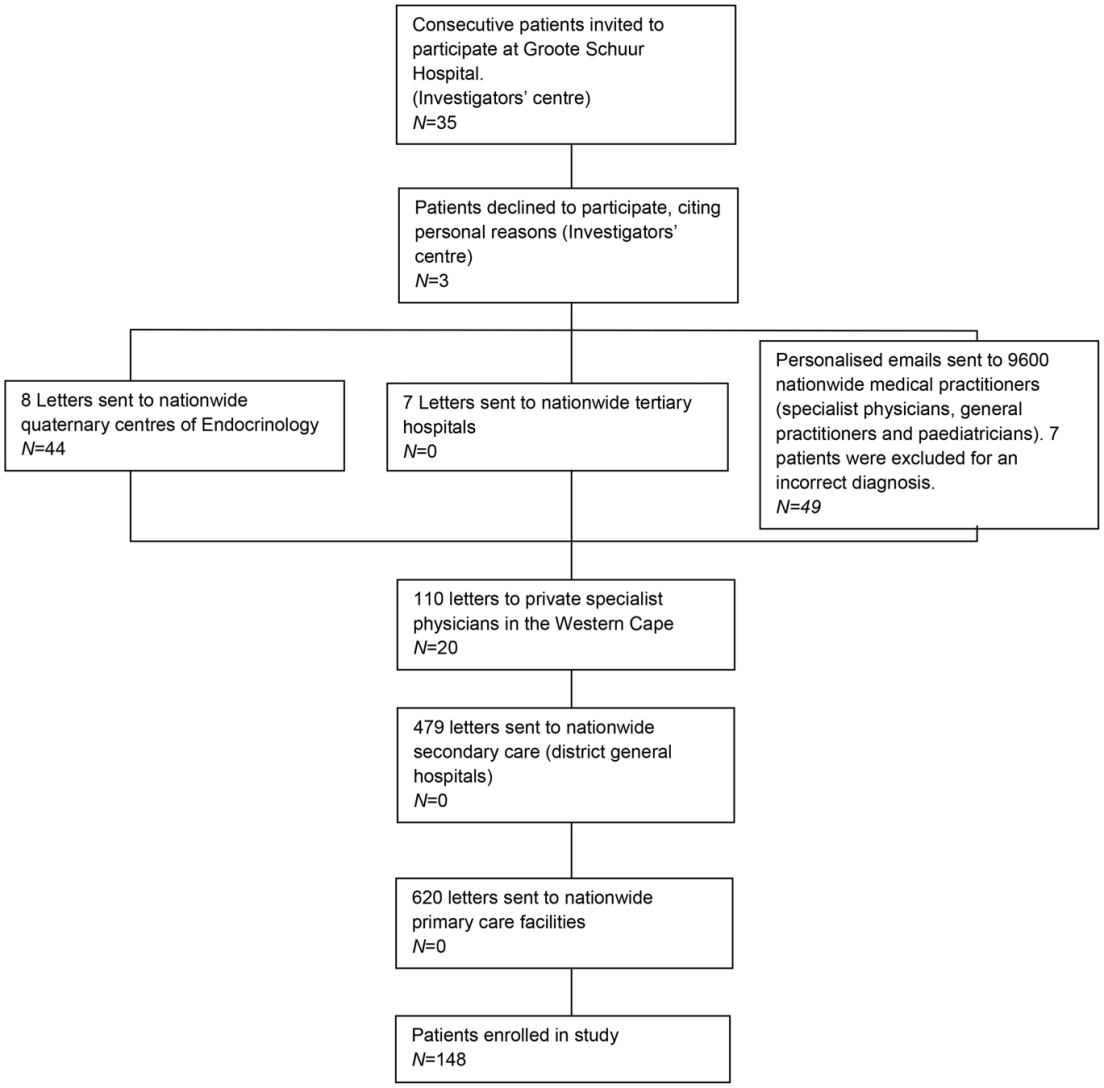
Flow diagram illustrating the pattern of referrals from each of the clinical service tiers for the years 2005–2010. As far as is known only 3 patients declined to participate, citing personal reasons. *N*: numbers of patients enrolled. Seven patients were excluded as 2 had a normal ACTH stimulation test, 2 had secondary hypoadrenalism, 1 had a bilateral adrenalectomy for Cushing’s disease, and 2 had suppression of the hypothalamic-pituitary adrenal (HPA) axis, related to previous steroid use for another indication.

There were 161 patients who were referred for enrolment in the South African Addison’s study. Seven patients with an original label of Addison’s disease were excluded: two had a normal ACTH stimulation test, two had secondary hypoadrenalism, one had a bilateral adrenalectomy for Cushing’s disease, and two had suppression of the hypothalamic-pituitary-adrenal (HPA) axis, related to previous steroid use for another indication. Three patients declined to participate, citing personal reasons and a further three patients were too late to be enrolled in this observational study. Thus, a total of 148 patients were enrolled in the study and we had obtained 92 (62.1%) transcripts describing the original clinical presentation. The referral pattern for the patients enrolled in the study is illustrated in figure 1.

### Demographic Data

The demographic data are shown in Table1. The majority of the patients lived in urban areas (87%), whereas isolated pockets of patients were identified in the northern part of the Western province, southern Cape and various districts of Kwazulu-Natal.

**Table 1:** Demographic data of 148 patients.

Variable	
Gender	
Males *N* (%)	57 (39)
Females *N* (%)	91 (61)
Age at enrolment years (IQR)	46.0 (32.0–61.0)
range (years)	2.8–88.0
Age at initial diagnosis years (IQR)	34.0 (20.0–45.0)
range (years)	0.02–77.0
Ethnicity	
Whites *N* (%)	97 (66)
Mixed Ancestry *N* (%)	34 (23)
Asian *N* (%)	5 (3)
Black *N* (%)	12 (8)
Foreign ancestry^1^ *N* (%)	51 (34)
Resident in urban areas *N* (%)	128 (87)

*N*: number.

IQR: interquartile range.

1: First or second degree foreign relative from the United States of America or Europe.

### Urban Prevalence

While the overall prevalence for this nationwide study was calculated at 3.1 per million, the urban specific prevalence was determined. The Western Cape demonstrated urban prevalence was 13.6 per million, followed by the Eastern Cape 4.7 per million, Gauteng 2.5 per million, Free State 1.8 per million, Northern Cape 1.7 per million and Kwazulu-Natal 0.58 per million.

### Clinical Characteristics

The most common presenting sign was hyperpigmentation (76%), followed by symptoms related to the gastrointestinal system, and with nausea and vomiting being reported by more than 40% of the participants (Table 2). Weight loss was reported in 25%. A significant number reported having suffered loss of consciousness at presentation (20% 95% CI 14–28%), had a history of collapse (7%, 95% CI 2.9–11.1%) and suffered from shock (5%, 95% CI 1.5–8.5%). Any of these three features could be suggestive of an Addisonian crisis, but the presence of shock is most convincing for an Addisonian crisis. Backache occurred in 20%, while dizziness (11%) and salt craving occurred in 15%. The latter two symptoms are suggestive of mineralocorticoid deficiency, but dizziness may also be a feature of glucocorticoid deficiency. Non-specific symptoms such as malaise and lassitude were noted in 8% and 3% of the cohort were noted to have hypoglycaemia as their presenting symptoms. Thus, the true number of patients who presented with an Addisonian crisis may be higher than 5%.

**Table 2:** Addison’s disease: clinical presentation.

Presenting symptom	*N*	Proportion of the cohort (%)
Self-reported increase in skin pigmentation	112	76
Nausea	76	51
Vomiting	63	43
Weight loss	37	25
Abdominal pain	31	21
Loss of consciousness	30	20
Backache	29	20
Salt craving	22	15
Diarrhoea	21	15
Dizziness	16	11
Malaise/lassitude	12	8
History of collapse	10	7
Shock	7	5
Hypoglycaemia	4	3
Anorexia	4	3
Hypoglycaemia	4	3
Anorexia	4	3

*N* number:

The proportion of 148 Addison’s disease patients who manifested with any of these symptoms at presentation.

### Co-morbidity Reported at Enrolment

As seen from Table 3, hypertension, hypercholesterolaemia and type 2 diabetes mellitus were the most prevalent cardiovascular conditions. A prior history of pulmonary tuberculosis was the most common respiratory condition. There were numerous infrequently encountered co-morbid conditions (≤1%), such as inter-alia steatosis of uncertain aetiology, prosthetic cardiac valve replacement, pulmonary emboli and chronic obstructive pulmonary disease. Hypothyroidism, type 1 diabetes mellitus, premature ovarian failure, coeliac disease were the most common associated autoimmune conditions.

**Table 3:** Co-morbidity reported at enrolment.

System	Medical condition^Σ^	*n*/*N* (%)
Cardiovascular system	Hypertension	22/148 (15)
	Type 2 diabetes mellitus	9/148 (6)
	Hypercholesterolaemia	7/148 (5)
	Ischaemic heart disease	4/148 (3)
	Cerebrovascular disease	3/148 (2)
Respiratory system	Tuberculosis	11/148 (7)
Rheumatological	Osteoporosis	7/148 (5)
	Antiphospholipid syndrome	4/148 (3)
Other	Osteoarthritis	3/148 (2)
	type 1 diabetes mellitus	11/148 (7)

*n*: Number of patients found to have a co-morbid illness.

*N*: Total number of Addison’s subjects.

Σ: Excludes medical conditions occurring in less than 1% of the patients, for example, steatosis, prosthetic cardiac valve replacement, pulmonary emboli and chronic obstructive airways disease.

### Biochemistry at Diagnosis

There was adequate biochemistry for the diagnosis of Addison’s disease in 72% of the cases (Table 4). This was defined by the presence of at least one of suggestive electrolytes (hyponatraemia and hyperkalaemia), low morning cortisol and simultaneously elevated ACTH levels or an inadequate cortisol response to an ACTH stimulation test. The high proportion of patients (28%) with inadequate biochemical verification is likely to be due to the study design in that these data were obtained retrospectively, in some instances several years prior to being enrolled in the study. Although some patients had multiple biochemical data available, diagnostic ACTH stimulation tests were available in 38 (26%), early morning cortisol and simultaneously elevated ACTH levels in a further 52 (36%) and suggestive biochemistry in 15 patients (10%). The respective median basal and stimulated cortisol concentrations were 58 nmol/L 82 nmol/L, range 5.9 to 476 nmol/L. Concentrations of ACTH greater than 10.1 pmol/L were considered appropriately elevated for this range of cortisol, which corroborates the presence of primary hypoadrenalism. The 105 patients with adequate or suggestive biochemistry were compared to the 43 patients with insufficient biochemical verification data. The single difference identified was that more patients with an adequate or suggestive biochemistry presented with vomiting, compared with the subjects with insufficient biochemical verification (table 5).

**Table 4:** Biochemistry at initial diagnosis of Addison’s disease.^1^

	Reference range NHLS	*n*/*N* (%)	Missing data *n*/*N* (%)	Median	Inter-quartilerange	Range
Basal cortisol(nmol/L)**	171.0–536.0	89/148 (59)	59/148 (40)	58.0	23.8–114	0.0–398
Stimulated cortisol(nmol/L)**	>550.0	38/148 (26)	110/148 (74)	82.0	49.8–20.8	0–476
Plasma ACTH (pmol/L)**	1.0–10.1	50/148 (34)	98/148 (66)	376	178–973	12.2–1878
Serum Na (mmol/L)**	135.0–147.0	52/148 (35)	96/148 (65)	129.0	125–136	101–145
Frequency of hyponatraemia		35/52 (62)				
Serum K (mmol/L)**	3.5–5.3	49/148 (33)	99/148 (67)	5.2	4.5–5.8	3.8–8.4
Frequency of hyperkalaemia		17/49(35)				
Renin (mU/L)**	7.0–76.0	22/148(15)	126/148 (85)	62.0	24–470	12–5500
Frequency of hyperreninemia		11/22 (50)				
Aldosterone (pmol/L)**	110.0–860.0	23/148 (16)	125/148 (84)	30.9	25–139	0.09–344
Frequency of hypoaldosteronaemia		18/23 (78)				
Serum TSH (mIU/L)	0.35–5.5	50/148 (34)	98/148 (66)	2.43	1.07–7.83	0.01–100
Frequency of hypothyroidism		13/50 (26)				
Serum free T4 (pmol/L)	11.5–22.7	37/148 (25)	111/148 (75)	14.2	10.4–17.0	0.1–26.8

1: different assay methods were used for each of the different analytes, but are included together for comparison.

*n*: Number of patients identified with available biochemical parameter.

*N*: Total number of Addison’s patients.

Plasma ACTH plasma adrenocorticotrophic hormone.

Serum Na: Serum sodium.

Serum K: Serum potassium.

Serum TSH: Serum thyroid stimulating hormone.

Serum free T4: Serum free thyroxine.

** Laboratory investigations are not mutually exclusive.

NHLS: National Health Laboratory Services.

Reference ranges as offered by the National Health Laboratory Services of 2011.

Reference ranges likely differed as different assays were used in the diagnosis.

**Table 5:** Clinical characteristics of Addison’s patients with adequate or suggestive biochemistry compared to those with insufficient biochemical verification.

Clinical characteristics	Adequate or suggestive biochemistry	Insufficient biochemical verification	*p* - value
Number	105	43	
Age of enrolment years (IQR)	31.0 (35.0–62.0)	41.0 (21.5–54.5)	0.12
Age at initial diagnosis	34.0 (20.0–46.8)	30.0 (16.3–42.5)	0.26
Gender Female *N* (%)	67 (64)	24 (58)	0.46
Ethnicity *N* (%)			0.28
White ancestry	72 (69)	25 (56)	
Mixed ancestry	23 (22)	11 (26)	
Asian	4 (4.5)	1 (2)	
Black	6 (5.5)	6 (14)	
Foreign ancestry *N* (%)^1^	36 (34)	15 (35)	0.49
Presenting symptoms *N* (%)			
Pigmentation	77 (73)	31 (72)	0.72
Nausea	57 (54)	19 (44)	0.36
Vomiting	51 (49)	12 (28)	**0.02**
Weight loss	25 (24)	12 (28)	0.60
Abdominal pain	22 (21)	9 (21)	1.0
Backache	23 (22)	6 (14)	0.27
Loss of consciousness	23 (22)	7 (16)	0.60
Diarrhoea	18 (17)	3 (7)	0.18
Salt craving	16 (15)	6 (14)	0.84
Dizziness	10 (10)	6 (14)	0.43
Delay in diagnosis months (IQR)	6.0 (3.0–18.0	5.0 (2.0–13.0	0.28
Comorbidity of enrolment *N* (%)			
Hypercholesterolaemia	5 (5)	2 (5)	0.70
Type 2 diabetes mellitus	7 (7)	2 (5)	0.93
Hypertension	18 (17)	4 (9)	0.80
Ischaemic heart disease	3 (3)	1 (2)	0.71
Cerebrovascular disease	1 (0.7)	2 (2)	0.42
Tuberculosis	7 (7)	4 (9)	0.83
Replacement therapy			
total daily hydrocortisone dose mg (IQR)	20.0 (20.0–30.0)	25.0 (20.0–30.0)	0.91

*N*: number.

1: first or second degree foreign relative from the United States of America or Europe.

### Replacement Therapy

Most patients (76%) received a combination of fludrocortisone and hydrocortisone. Prednisone in combination with hydrocortisone was the next most common combination of steroid replacement administered (table 6). The median total daily hydrocortisone dose was 20 mg and the total median dose of hydrocortisone corrected for body surface area (hydrocortisone/m^2^) was 12.4 mg. In this cohort, 33% were receiving three daily doses, 52% were receiving two daily doses and 15% were receiving a single daily dose of glucocorticoid replacement therapy. In addition, 38% of patients reported having had at least one lifetime Addisonian crisis and 58% of patients did not wear any form of medical alert identification. The only patient who used dexamethasone as replacement therapy, was receiving a significantly greater glucocorticoid exposure than any of the other patients.

**Table 6:** Glucocorticoid replacement therapy in South African Addison’s disease patients.

Preparation	*n*/*N*(%)	Median daily dose (IQR)	Equivalent hydrocortisone dose/kg (IQR)	Equivalent hydrocortisone dose/m^2^ (IQR) [29]
Hydrocortisone	112/148 (76)	20.0 (20-30.0)	0.33 (0.25-0.44)	12.4 (10.3-16.9)
Cortisone acetate	3/148 (2.0)	25.0 (25.0-32.5)	0.53 (0.50-0.55)	19.7 (18.3-21.0)
Prednisone	10/148 (8)	8.75 (5.0-11.9)	0.42 (0.3-0.68)	16.6 (11.36-26.3)
Dexamethasone	1/148 (0.7)	5	1.31	62

*n*: Total number of patients identified using a specific form of glucocorticoid replacement therapy.

*N*: Total number of Addison’s patients enrolled in this analysis.

IQR: Inter-quartile range.

Hydrocortisone dose/m^2^: Total daily hydrocortisone dose, corrected for body surface area.

Hydrocortisone dose/kg: Total daily hydrocortisone dose corrected for body weight.

Missing data in 22/148 (15%), patients untraceable.

Equivalent doses derived from Meikle AW and Tyler FH. Potency and duration of action of glucocorticoids. Am J of Med 1977;63;200 [29].

## Discussion

There are numerous cohort studies of Addison’s disease in the literature, but this is the only one conducted in sub-Saharan Africa and it can be considered to represent a large cohort as 148 patients were enrolled [5], [12], [13]. Although an extensive effort was made to identify every person with Addison’s disease in South Africa, it is possible that some patients may not have been captured. The cohort was generated from referrals of all tiers of hospital services in South Africa, where no databases for this disease are kept, except within the domains of the medical insurance companies. In most cohort studies, the participants were exclusively drawn from academic centres, with the exception of two reports [7], [14]. In this study and that of Soule, loss of consciousness and confusion were recorded as presenting symptoms, which suggests that patients with Addison’s disease may present in a more advanced state of ill-health, compared to their first world counterparts [11]. For example, none of the 216 patients in a German cohort appeared to manifest with an overt Addisonian crisis [7].

Although we attempted to obtain a complete biochemistry dataset for each of the patients, this is not possible, because in many instances patients had been diagnosed with Addison’s disease up to 20 years prior to being enrolled in the study and early clinical and biochemical records of the initial admission were not available. Biochemical data compatible with the diagnosis were available for at least 72% of the patients enrolled. Nevertheless, there is no reason to doubt the diagnosis of primary hypoadrenalism, since the diagnosis was made by experienced specialist physicians, paediatricians, and endocrinologists and we verified that the clinical picture at presentation was compatible with this diagnosis. The clinical characteristics were similar in the groups of patients with adequate biochemistry in the group with an adequate biochemistry verification data.

Based on the data from Lovas et al and Ten et al [3], [4], the prevalence of Addison’s disease is estimated to vary from 39 to 117 per million, but it has been recorded as high as 144 per million [5]. Even using conservative estimates, this is considerably higher than the 3.1 per million found in the current study. It is likely that this cohort may have not included all cases of Addison’s disease as the majority of the participants were expected to be black African, with similar numbers of white mixed ancestry patients and a small number of Asian patients. The predominantly white patients in this study may reflect that the evolution of Addison’s disease is dependent on certain HLA frequencies, genetic variations or environmental factors, creating favourable conditions that promote a greater prevalence among whites [15]. As 87% of the cohort was found to be resident in urban areas, we examined the urban specific prevalence, which varied from 13.6 per million to 0.58 per million, which is still considerably lower than reported in Western studies. The wide range of urban specific prevalence may reflect that the Western Cape has comparatively has a more favourable doctor to patient ratio. See www.hst.org.za accessed November 2012. It may also reflect that the researchers originated from the Western Cape and had the greatest interest in finding cases. The highest prevalence in the Western Cape may also reflect the minor differences in ethnic make-up, compared to the remaining provinces. See www.statssa.gov.za accessed November 2012.

Another possible explanation is that patients with Addison’s disease may die with the disease unrecognised. Addison’s disease is regarded as one of the great mimickers of medicine. Although hyperpigmentation is a significant clinical feature in whites, it may be more subtle in darkly pigmented races, resulting in the diagnosis being overlooked [11], [16], [17]. The disease may also be unrecognised due to the challenged health-care system, with some facilities having only 7% of the required number of doctors [18].Enhancing awareness of Addison’s disease may be achieved by providing annotations on chemical pathology reports alerting clinicians to the possibility that primary hypoadrenalism may coexist with compatible biochemistry. This should be tempered by the fact that recent interpretive commenting was diverse, incorrect in some cases and potentially misleading [19]. Awareness of Addison’s disease should be raised among medical students.

Analogous to the explanation of the low incidence of Addison’s disease in rural settings is a lower incidence of type 1 diabetes mellitus in rural Africa, where the ratio of medical practitioners to population is considerably less than the cities [20]. The distribution example systemic lupus erythematosis (SLE) has also been found to vary, even with in an urban district, suggesting that geographical factors may play a role in modifying an autoimmune process [21]. Taken together, the few black patients enrolled in our study may reflect the sub-optimal health delivery in rural areas and the particular challenge in making this diagnosis in pigmented races.

In some respects, this cohort is congruent with the findings of previous studies, for example multiple authors have confirmed the preponderance of women in the cohorts [12], [14] and the age of diagnosis is similar to many studies of adult Addison’s disease [13], [22], [23]. As can be anticipated, the age of onset in paediatric Addison’s disease population differs substantially, as the median age of onset was 7.7 years in the study by Simm et al [24]. The constellation of hyperpigmentation, nausea, vomiting and weight loss should suggest Addison’s disease as attested to by at least three other studies [7], [11], [25].

It is concerning that a significant number (58%) of patients did not wear any formal medical identification in this study, which is considerably higher than the 40% of type 1 diabetic children, aged between six years and 17 years of age, who did not wear medical alert identification that indicated that they were diabetic and required insulin [26].

The patients in the study appeared to have significant comorbidity, but it remains unclear whether this is greater than that experienced by the general population in South Africa. On the other hand, recent data from the United Kingdom indicate that aside from expected coexistent autoimmune disease, 65% of patients with Addison’s disease had an elevated body mass index (BMI) of greater than 25 kg/m^2^, 65% of the total cholesterol of greater than 5 mmol/L, 17.9% is establish spinal osteoporosis and 53.5% of spinal osteopenia, corroborating the fact that Addison’s patients are burdened by comorbidity [27].

Mortality has not been assessed in patients with Addison’s disease in South Africa, but in Sweden, this has been examined by reviewing death registers. Cardiovascular causes of mortality, especially ischaemic heart disease were twice as common as the background population. Malignancy with no specific predilection for a specific sub-type and infectious diseases were also considerably greater than the background population. In the same study, diabetes irrespective of the type conferred an almost quadruple mortality in patients compared to the background population [28].

The current study of South African Addison’s disease has several weaknesses. As it includes a cross-sectional analysis of the clinical characteristics of Addison’s disease, the diagnosis in many instances was made several years previously. These, together with the lack of uniform protocols for evaluating Addison’s disease and incomplete clinical notes in a diversity functioning health-care system, probably contributed to the incomplete available data. It has been noted that the prevalence of Addison’s disease in South Africa is considerably lower than in Western countries. Access to health care in rural regions could have accounted for this low prevalence. Moreover, the symptoms reported at enrolment were subject to recall bias as the diagnosis was made 12 years previously. Another potential weakness is that Addison’s patients who did not survive an acute Addisonian crisis before being enrolled in the study could have resulted in the prevalence being underestimated.

This large cohort from sub-Saharan Africa contained small proportions of black and Asian participants, which do not reflect the overall demographics of the country. The overall prevalence of Addison’s disease is considerably lower than Western countries and the urban specific prevalence is also reduced, likely due to under diagnosis. As in the rest of the world, the usual constellation of symptoms and signs suggest Addison’s disease, but it patients appear to present in an advanced state of ill-health. Raising awareness of this highly treatable condition is important and is potentially a life-saving measure.
